# Rapidly Progressive Lung Cysts and Pleural Effusion: A Case Report

**DOI:** 10.1155/2011/790274

**Published:** 2011-09-08

**Authors:** Diana Olsen, Charlene Molloy, P. S. Sriram

**Affiliations:** Division of Pulmonary, Critical Care, and Sleep Medicine, University of Florida, Gainesville, FL 32608, USA

## Abstract

Angiosarcoma is a rare but highly malignant tumor arising from vascular endothelial cells. Angiosarcoma commonly arises from the heart, liver, breast, and skin including the scalp. Angiosarcoma metastasizing to the lungs can present as either pneumothorax, hemothorax, or pleural effusions. They can rarely present as rapidly enlarging thin-walled pulmonary cysts. A review of the literature is included.

A 73-year-old Asian man was admitted to the hospital with a 2-month history of progressive dyspnea and scant hemoptysis. He denied fevers, weight loss, or night sweats. He had diabetes, hypertension, and coronary artery disease and had 3 vessel CABG a year prior. Six months prior to presentation he was diagnosed with scalp angiosarcoma and underwent wide local excision followed by localized radiation. He was also diagnosed to have high grade transitional cell bladder cancer at the same time. He was a 50-pack-year ex-smoker. He was a farmer who had lived in Central America for many years in the 1980s and frequently visited South Asia. 

His room air oxygen saturation was 91%. His physical examination was normal except for decreased breath sounds in the left base and crackles at the right base. There were no skin lesions. Blood count and chemistry were normal. A chest radiograph revealed a new left sided pleural effusion (Figure 1). Chest CT showed a moderate sized left pleural effusion and numerous thin-walled cystic lesions predominantly in the right lung which were new in comparison to a CT scan 6 months ago (Figure 2). Serum ANA, rheumatoid factor, c-ANCA, p-ANCA, and HIV serologies were negative. Thoracentesis revealed a serosanguinous fluid with glucose of 87 mg/dL, protein of 3.8 g/dL, LDH of 381 U/L (serum protein 6.4 g/dL and LDH 437 U/L), and WBC of 462/*μ*L with 94% eosinophils. Pleural fluid cytology on two separate occasions was negative for malignancy. Bronchoscopy with transbronchial biopsies of the right lower lobe showed nonspecific interstitial pneumonia with occasional eosinophils. A CT scan of the chest obtained 3 weeks later showed marked increase in number and size of the cystic lesions. Patient underwent medical thoracoscopy which revealed several lesions on the parietal pleura (Figure 3). Thoracoscopic biopsies of the parietal pleural lesion showed pleomorphic cells with abundant pale cytoplasm, hyperchromatic nuclei, and abundant mitotic figures. These cells were CD 31 and CD 34 positive and negative for calretinin, cytokeratin, CEA (carcinoembryonic antigen), TTF1 (thyroid transcription factor), and EMA (epithelial membrane antigen) (Figure 4). A final diagnosis of metastatic angiosarcoma was made. Morphologically it was identical to his prior scalp tumor. 

Angiosarcoma is a rare but highly malignant tumor arising from vascular endothelial cells. They are extremely rare and comprise less than 2% of all sarcomas [1]. Angiosarcoma affects every organ but most commonly arises from the heart, liver, breast, and skin including the scalp. These tumors are classified into 4 categories: cutaneous angiosarcoma without lymphedema, cutaneous angiosarcoma with lymphedema, angiosarcoma of the breast, and angiosarcoma of the deep soft tissue [2]. They have a male predilection and generally affect the elderly. While primary pulmonary or pleural angiosarcoma is extremely rare with fewer than 10 reported cases, lung is one of the most common sites of metastasis. Other sites include the liver and lymph nodes [3]. Metastatic angiosarcoma to the lungs most frequently originates from the scalp and face. A review of Japanese autopsy data of 95 angiosarcoma cases revealed that the scalp was the primary site in almost 35 percent of cases. In this series angiosarcoma originating from the scalp often presented with pneumothorax, hemothorax, or atelectasis. Pneumothorax was only noted with scalp angiosarcoma compared to nonscalp angiosarcoma [4]. It is believed that angiosarcoma from the scalp metastasizes to the subpleural or pleural surfaces which subsequently undergo necrosis resulting in a pneumothorax. Rarely patients can present with spontaneous hemothorax or hemorrhagic pleural effusions; the majority of them are bilateral [2, 5]. Pleural fluid cytology is seldom diagnostic; however, if malignant cells are present, immunocytochemical analysis for endothelial markers CD 31, CD 34, von Willebrand factor related antigen (VIII-RA), and *Ulex europaeus* agglutinin-1 (UEA-1) can confirm the diagnosis of angiosarcoma [6, 7]. It is speculated that patients develop hemothorax due to bleeding from metastatic pleural angiosarcoma or due to rupture of peripheral lung metastatic foci into the pleural space [2]. Patients who present with a hemothorax have an extremely poor prognosis. Most of them die within a few months of diagnosis. 

There have been rare case reports of patients presenting with thin-walled pulmonary cysts that develop and enlarge over several months [8]. These cysts rupture resulting in a pneumothorax. It is thought that these cystic lesions occur as a result of partial obstruction of the small airways by tumor causing a ball-valve effect and resultant hyperinflation of the alveoli.

Clinical manifestations of angiosarcoma with metastasis to the lung can include dyspnea, cough, chest pain, and hemoptysis [1]. Often patients are asymptomatic, and abnormality is incidentally noted on chest radiograph. On chest radiograph and CT scan metastatic pulmonary angiosarcoma can present as diffuse alveolar infiltrates consistent with pulmonary hemorrhage, spontaneous pneumothorax, pleural effusion, thin-walled cystic cavities, and nodular lesions [3]. Patients with metastatic pulmonary angiosarcoma have an extremely poor prognosis. There is no effective therapy, and most patients die within several months of diagnosis. 

On histology, tumor cells are pleomorphic with abundant pale cytoplasm, hyperchromatic nuclei, and abundant mitotic figures. These cells are often arranged to form neoplastic vascular channels. While histopathology can be similar in Kaposi's sarcoma, pulmonary capillary hemangiomatosis and metastatic spindle cell carcinomas, immunohistochemistry can help confirm the diagnosis of angiosarcoma. Tumor cells are often positive for vimentin, endothelial markers CD 31, CD 34, von Willebrand factor related antigen (VIII-RA), and *Ulex europaeus* agglutinin-1 (UEA-1). They are negative for epithelial (cytokeratin) and mesothelial markers (calretinin) [3]. 

We believe that cystic lesions in our patient were a result of metastatic deposits in the lung parenchyma. Our patient successfully underwent talc poudrage during medical thoracoscopy. He was discharged home to follow up with oncology. However, his overall condition continued to deteriorate and he died less than a month following diagnosis.

In summary, angiosarcoma is a rare but highly malignant vascular endothelial tumor that commonly metastasizes to the lung. It can present as rapidly enlarging cystic pulmonary lesions and pleural effusion. Immunohistochemistry can help confirm the diagnosis.

## Figures and Tables

**Figure 1:**
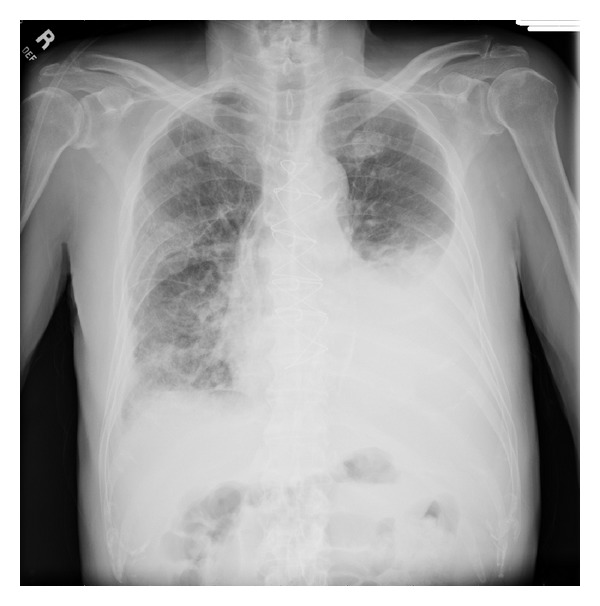
Chest radiograph showing moderate size left pleural effusion.

**Figure 2:**
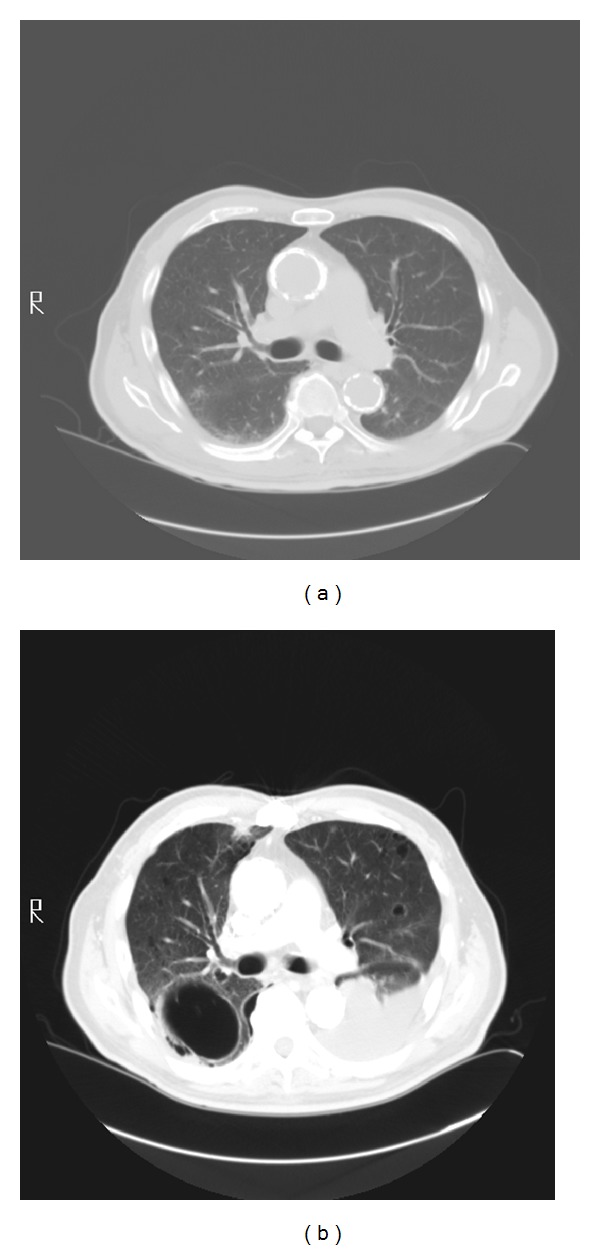
CT scans of the chest at 6-month interval showing a new left pleural effusion and a large thin-walled cystic lesion in the right lung.

**Figure 3:**
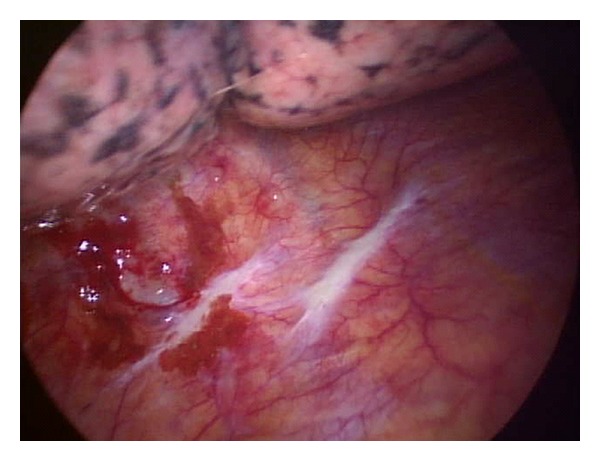
View of left thoracic cavity on medical thoracoscopy demonstrating tumor implants on the parietal pleural surface.

**Figure 4:**
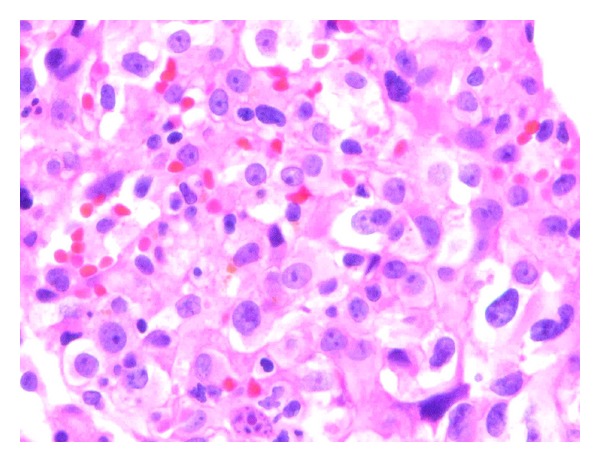
Photomicrograph of thoracoscopic biopsy specimen (hematoxylin-eosin, original × 100) demonstrating pleomorphic endothelial cells with pale cytoplasm, hyperchromatic nuclei, and mitotic figures.
